# Consensus and conflict cards for metabolic pathway databases

**DOI:** 10.1186/1752-0509-7-50

**Published:** 2013-06-26

**Authors:** Miranda D Stobbe, Morris A Swertz, Ines Thiele, Trebor Rengaw, Antoine HC van Kampen, Perry D Moerland

**Affiliations:** 1 Bioinformatics Laboratory, Academic Medical Center, University of Amsterdam, P.O. Box 22700, Amsterdam 1100 DE, the Netherlands; 2 Biosystems Data Analysis, Swammerdam Institute for Life Sciences, University of Amsterdam, Science Park 904, Amsterdam 1098 XH, the Netherlands; 3 Luxembourg Centre for Systems Biomedicine, University of Luxembourg, 7, avenue des Hauts-Fourneaux, Esch-sur-Alzette L-4362, Luxembourg; 4 Genomics Coordination Center, University Medical Center Groningen & University of Groningen, P.O. Box 30001, Groningen 9700 RB, the Netherlands; 5 Netherlands Bioinformatics Centre, Geert Grooteplein 28, Nijmegen 6525 GA, the Netherlands; 6 Netherlands Consortium for Systems Biology, University of Amsterdam, P.O. Box 94215, Amsterdam 1090 GE, the Netherlands; 7 Current address: Institute for Research in Biomedicine (IRB Barcelona), c/Baldiri Reixac 10, Barcelona 08028, Spain

**Keywords:** Metabolic network, Consensus, Community support, Human, Pathway database

## Abstract

**Background:**

The metabolic network of *H. sapiens* and many other organisms is described in multiple pathway databases. The level of agreement between these descriptions, however, has proven to be low. We can use these different descriptions to our advantage by identifying conflicting information and combining their knowledge into a single, more accurate, and more complete description. This task is, however, far from trivial.

**Results:**

We introduce the concept of Consensus and Conflict Cards (C_2_Cards) to provide concise overviews of what the databases do or do not agree on. Each card is centered at a single gene, EC number or reaction. These three complementary perspectives make it possible to distinguish disagreements on the underlying biology of a metabolic process from differences that can be explained by different decisions on how and in what detail to represent knowledge. As a proof-of-concept, we implemented C_2_Cards^Human^, as a web application http://www.molgenis.org/c2cards, covering five human pathway databases.

**Conclusions:**

C_2_Cards can contribute to ongoing reconciliation efforts by simplifying the identification of consensus and conflicts between pathway databases and lowering the threshold for experts to contribute. Several case studies illustrate the potential of the C_2_Cards in identifying disagreements on the underlying biology of a metabolic process. The overviews may also point out controversial biological knowledge that should be subject of further research. Finally, the examples provided emphasize the importance of manual curation and the need for a broad community involvement.

## Background

Metabolic pathway databases have proven very valuable for a wide range of applications, varying from the analysis of high-throughput data to *in silico* phenotype prediction. In the past decade the number of pathway databases has grown markedly, providing extensive descriptions of the metabolic network for an increasing number of organisms [1,2]. The metabolic networks of several key organisms, for example, *S. cerevisiae* and *H. sapiens,* are even described in multiple databases. A comparison of two yeast networks showed, however, that the two agreed on only 36% of their reactions [3]. Similarly, five pathway databases describing the human metabolic network agreed on only 3% of the 6968 reactions they jointly contain [4]. Given that these databases aim to represent the metabolic capabilities of the same organism, the level of agreement is much lower than one might expect and hope for. There are several explanations for the observed lack of consensus. These include the different ways in which the networks have been built, their manner of curation, and a different interpretation of literature [5]. The comparison of Stobbe *et al.*[4] also revealed large differences in the breadth and depth of the coverage the five human metabolic networks have.

The advantage of having several descriptions of the metabolic network for the same organism is that they offer different views on the same biological system and thus can reveal controversial biological knowledge. In addition, the databases each have a particular focus and its curators have specific fields of expertise. Therefore, each database may provide complementary pieces of the puzzle of the complete metabolic network. These observations have motivated, still ongoing, efforts to consolidate the different networks for the same organism and to build consensus metabolic networks using a largely manual approach [3,6,7].

Combining all the knowledge on the metabolic network contained in the various pathway databases and identifying conflicting information is, however, far from trivial. Retrieving all required information from multiple databases is in itself already a cumbersome task. One reason that makes it challenging to identify instances where pathway databases do not agree on the underlying biology of a metabolic process are the different decisions made by each of the databases on how to represent knowledge [4,8]. For example, a particular difference may be simply explained by the different levels of granularity with which metabolic processes are described by each database, instead of a fundamentally different biological insight. Secondly, it remains a challenge to determine whether databases refer to the same gene or the same metabolite. Thirdly, the definition of a pathway also differs per database, which makes it nearly impossible to compare the networks on a smaller scale, *i.e.*, per pathway. Fourthly, the larger the number of pathway databases considered, the more difficult it is to identify the consensus and the conflicts. Recently, algorithms have been proposed to semi-automatically merge two descriptions of the metabolic network of the same organism [9,10]. These approaches mainly address the challenge of matching metabolites, partly via interactions with the user. The core of their resulting merged description consists of reactions that can be found in both networks. Integrating more than two descriptions will, however, significantly reduce the size of the core and limit its utility [4]. The merged description also contains reactions that could not be (exactly) matched and are therefore unique to one of the descriptions. Such an approach will, however, neither resolve the conflicting information between databases nor filter out erroneous information. Furthermore, the semi-automatic approaches do not explicitly address all issues mentioned above. For example, conflicts due to differences in granularity are not taken into account. While semi-automatic approaches generate a useful scaffold for a consensus network, the resulting description still requires extensive manual curation.

Altogether, the issues described above make the construction of a single, more accurate, and more complete network based on the pathway databases available a laborious and largely manual process [6]. Moreover, it is an ongoing process, as new knowledge continues to become available both in the scientific literature and in pathway databases.

To more easily visualize the opinion of multiple pathway databases, we introduce the concept of Consensus and Conflict Cards (C_2_Cards). C_2_Cards combine the knowledge from multiple pathway databases for a specific target organism. A C_2_Card can be centered at a single gene, Enzyme Commission (EC) number or reaction of interest and gives a concise overview of what the databases do or do not agree on with respect to the entity the C_2_Card is centered at. These three perspectives offer complementary views on the knowledge contained in the pathway databases. Importantly, using these perspectives disagreements caused by a different decision on how and in how much detail to represent knowledge can be identified. C_2_Cards can be used to assist reconciliation efforts and make users of pathway databases more aware of the exact differences that currently exist between databases.

As a proof-of-concept, we implemented C_2_Cards^Human^ (http://www.molgenis.org/c2cards), which combines the knowledge of the following five frequently used human pathway databases: the Biochemically, Genetically and Genomically structured (BiGG) knowledgebase [11] (*H. sapiens* Recon 1 [12]), the Edinburgh Human Metabolic Network (EHMN) [13], HumanCyc [14], and the metabolic subsets of the Kyoto Encyclopedia of Genes and Genomes database (KEGG) [15] and Reactome [16]. Below, we first give an overview of the various features of the C_2_Cards, the combined strength of the three perspectives, and how C_2_Cards can aid in the curation of gene and metabolite identifiers. Next, we describe several case studies illustrating the potential of the C_2_Cards in identifying conflicts between pathway databases. Finally, we discuss the next steps to be taken in curating metabolic networks.

## Results

**Figure 1:**
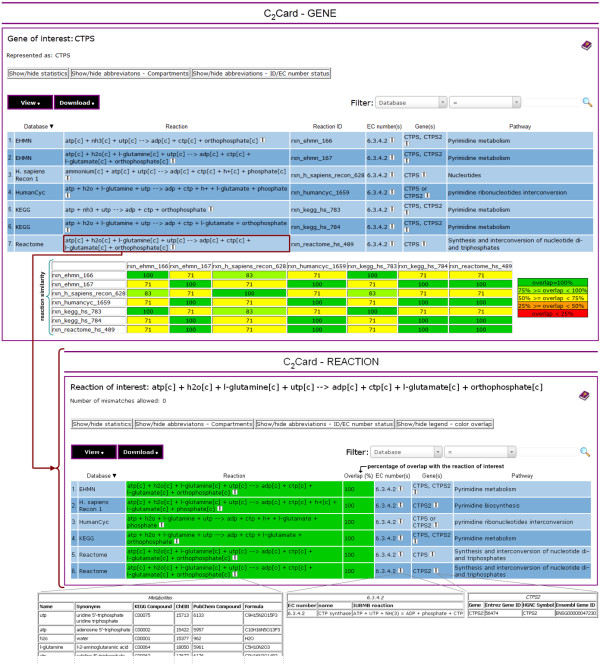
**Examples of two C**_**2**_**Cards.** C_2_Card centered at the *CTPS* gene (top) and the C_2_Card retrieved by clicking on the reaction of Reactome in the C_2_Card centered at the *CTPS* gene (bottom). Each C_2_Card consists of a table in which each row contains the following basic elements: a reaction and the EC number(s), gene(s) and pathway linked to it in one of the pathway databases. One can switch perspective by clicking on any of the elements in the table. For additional information provided by the pathway databases, *e.g.*, pathway visualizations and literature references, a direct link is provided to the original entry of the reaction in the pathway database. The second core ingredient of a C_2_Card is that each card explicitly shows the similarity of the reactions displayed on it. The percentage of overlap between reactions is indicated and relevant cells are colored according to the degree of overlap. Information on the IDs assigned to the metabolites and genes by a pathway database is shown by clicking on the **i** icon. For EC numbers the reaction and name linked to it by NC-IUBMB are shown.

Each C_2_Card provides an overview of the knowledge of multiple pathway databases from the perspective of a specific gene, EC number or reaction of interest. A C_2_Card answers the basic question of which databases contain the entity of interest. Importantly, each card provides a concise overview of what the databases do and do not agree on with respect to the entity of interest. The core component of a C_2_Card is a table in which each row contains the following basic elements: a reaction and the EC number(s), gene(s) and pathway linked to it in one of the pathway databases (Figure 1). If the information is available, complexes and isozymes are indicated by means of Boolean operators (see Materials and methods). Any of the aforementioned elements may be missing, except for the entity on which the C_2_Card is centered. By focusing on these basic elements, the overviews remain compact. For additional information provided by the pathway databases, *e.g.*, pathway visualization and literature references, a direct link is provided to the original entry of the reaction in the pathway database. The second core component of a C_2_Card is that each card explicitly indicates the similarity of the reactions displayed on it. Similarity is indicated either between all pairs of reactions (gene and EC number perspective; Figure 1) or with respect to the reaction of interest (reaction perspective; Figure 1). Here, reaction similarity is defined as the percentage of metabolites found in both reactions (see Materials and methods). The strengths of each of the three perspectives are discussed in more detail below.

### Three complementary perspectives

C_2_Cards offer three complementary perspectives (gene, EC number, reaction) on the knowledge contained in the pathway databases. Each perspective can answer various types of questions, accommodating the different interests one may have. Importantly, the three perspectives can be used to identify and complement information missing in one (or more) of the pathway databases using the knowledge from the other pathway databases.

#### Gene perspective

The ’gene perspective’ shows for each of the pathway databases, which metabolic functions the product of a gene has, as indicated by the reaction(s) and EC number(s) linked to it. This perspective may also answer the question whether other genes, either encoding isozymes or components of the same complex, are linked to the same reaction.

#### EC number perspective

The ’EC number perspective’ shows on which elements linked to the EC number the pathway databases (dis)agree for a specific type of conversion. It may also reveal possible alternative substrates, which is one of the sources of conflict between metabolic pathway databases [4]. The C_2_Card centered at the EC number 1.1.1.35 (3-hydroxyacyl-CoA dehydrogenase) provides an example of this scenario (Additional file 1). For example, EHMN has 62 unique reactions linked to this EC number while both HumanCyc and Recon 1 only have two unique reactions. The EC number perspective can also be used to answer the question which genes encode for an enzyme with the specified enzymatic function, according to each database.

#### Reaction perspective

The ‘reaction perspective’ provides a compact overview of which gene(s) and EC number(s) are linked to a reaction of interest in each pathway database. This perspective can assist in resolving a commonly occurring gap in reconstructions of the metabolic network, namely cases in which the gene product catalyzing a known metabolic reaction is missing [17]. The reaction perspective (and also the EC number perspective) can be used to find possible candidates for a missing gene in a particular database or reveal that the gene is missing in all pathway databases.

**Figure 2:**
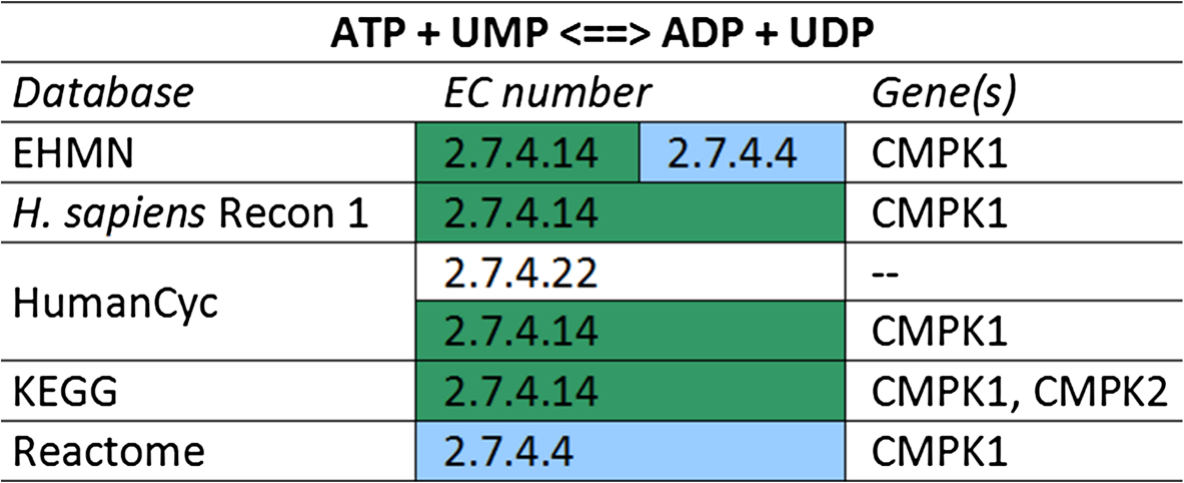
**Excerpt of the C**_**2**_**Card centered at the reaction ‘ATP + UMP <==> ADP + UDP’.** Different EC numbers linked to the same reaction and gene, which illustrates the difference in enzyme activity assigned to the product of the CMPK1 gene. Matching EC numbers have the same color.

By clicking on any of the entities shown in a C_2_Card one can easily switch perspective. Furthermore, each C_2_Card is opened in a new window to enable a simultaneous view of the C_2_Cards of a linked triple of a reaction, EC number, and gene from different viewpoints. Using all three perspectives is essential to get a complete picture of what the databases do or do not agree on. The EC number perspective can, for example, neither fully replace the gene perspective nor the reaction perspective, as illustrated by the example in Figure 2. An EC number does not uniquely identify a reaction or an enzyme. As the example shows, the pathway databases linked different EC numbers to the same reaction. Furthermore, in this case the databases either do not agree on the substrate specificity of the gene product, or curators assigned the EC number based on the reaction instead of the functionality of the gene product (Table 1). Finally, in the C_2_Cards application one can also cast a wider net when querying for an EC number by allowing a mismatch on the fourth number of an EC number. In contrast to the first three numbers, the last number does not indicate a specific subclass of enzymes and only serves to distinguish enzymes with different substrate specificities.

**Table 1:** Definition of EC numbers in NC-IUBMB

**EC number**	**Enzyme name**	**Reaction as defined by NC-IUBMB**
2.7.4.4	Nucleoside-phosphate kinase	ATP + nucleoside phosphate =
		ADP + nucleoside diphosphate
2.7.4.14	UMP/CMP kinase	(1) ATP + (d)CMP = ADP + (d)CDP
		(2) ATP + UMP = ADP + UDP
2.7.4.22	UMP kinase	ATP + UMP = ADP + UDP

The enzyme name and reaction(s) linked to the EC numbers of Figure 2 by the Nomenclature Committee of the International Union of Biochemistry and Molecular Biology (NC-IUBMB). The information of NC-IUBMB is available in a C_2_Card for each EC number that is part of the overview (see Figure 1). Note that the EC number 2.7.4.22 is unlikely to be correct in this case as NC-IUBMB indicates that this particular enzyme is the prokaryotic variant of the enzyme linked to EC number 2.7.4.4.

#### Dealing with conceptual differences

**Figure 3:**
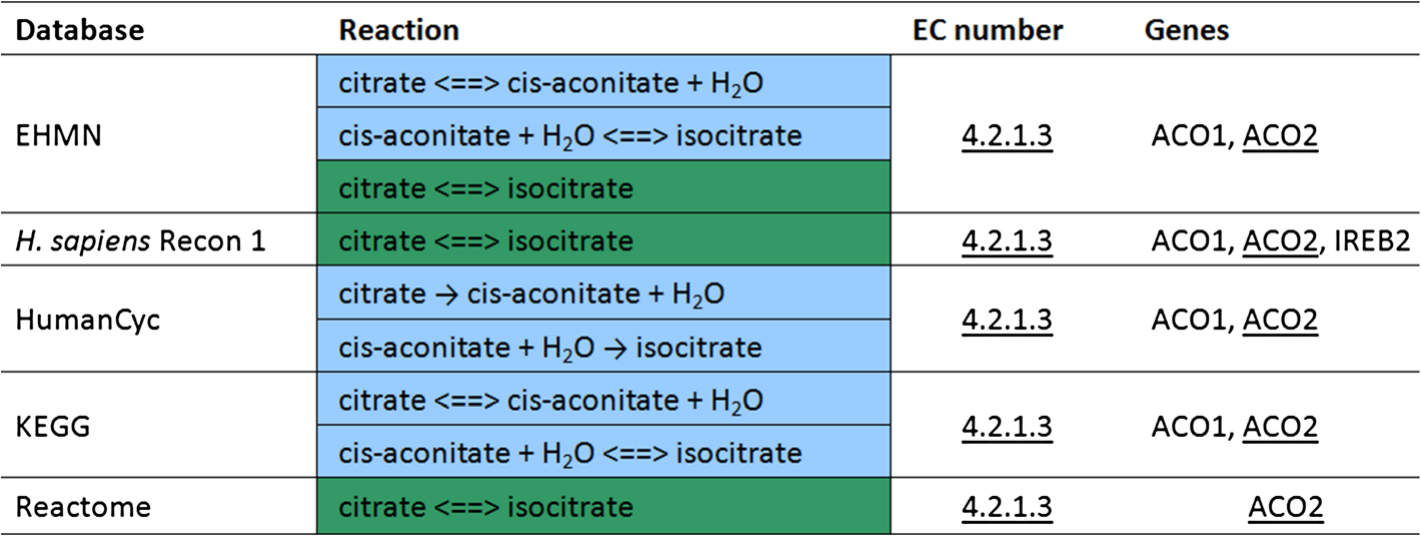
**Excerpt of the C**_**2**_**Card centered at the EC number ‘4.2.1.3’ (aconitate hydratase).** Conversion of citrate into isocitrate (part of the TCA cycle) in one (green) or two steps (blue). The EC number and gene on which all five databases agree are underlined.

Combining different perspectives also offers a way to side-step differences that do not reflect a true disagreement on the underlying biology such as the difference in the level of detail with which a metabolite or a conversion is described. Since such a difference will generally not affect the gene or EC number that is assigned to a reaction, these differences can be revealed using the gene or EC number perspective. One example is that some databases may provide the specific form of a metabolite, *e.g.*, α-D-glucose or β-D-glucose, while in other databases the more general form is used, D-glucose in this case. A possible motivation for database curators to choose the general version is that in an experiment the distinction between two isomers may be difficult to make. A second example is that one database may choose to describe a biochemical conversion in a single reaction using generic metabolites, like ‘a long chain alcohol’, versus multiple reactions with more specific examples of metabolites, *i.e.*, ‘hexadecanol’ and ‘octadecanol’ instead of ‘a long chain alcohol’, in another database. The gene or EC number perspective can be used to uncover such a difference. A third example is that the number of steps used to describe a biochemical process may differ, which will prevent a perfect match on reaction level as well. Note, however, that this difference in level of detail may not always be a conceptual difference, but could also be due to a disagreement on the underlying biology. This commonly occurring difference in the number of intermediate steps can be revealed via the gene or EC number perspective as well (Figure 3).

### Gene and metabolite identity

Next to exploring the genes, EC numbers, and reactions contained in the pathway databases, as described above, C_2_Cards can also be of direct use in curating the identifiers (IDs) assigned to the genes and metabolites by the pathway databases. Identifiers are essential for the unambiguous identification of genes and metabolites across multiple resources and enable linking experimental data to the metabolic network. For each gene and metabolite a C_2_Card provides the identifiers assigned to them by the pathway databases (see Figure 1, and Materials and methods). Obsolete or transferred identifiers are explicitly indicated. For genes the HUGO Gene Nomenclature Committee (HGNC) symbol is provided and for metabolites their name and synonyms. If available in a pathway database, two structural IDs (InChI and SMILES) and the chemical formula are also shown for a metabolite. The information on the identifiers helps to reveal cases where the assignment of identifiers to a metabolite or gene can be improved. Firstly, it can uncover metabolites that completely lack an ID in one or more pathway databases. Secondly, ID information can also help to identify cases where pathway databases assigned IDs from different gene and metabolite databases to the same entity. This can be used to propose additional identifiers for that particular gene or metabolite, which may also facilitate matching between databases. Thirdly, it can reveal genes and metabolites to which a pathway database assigned multiple identifiers from the same genome or metabolite database, respectively. In summary, C_2_Cards can assist the considerable amount of manual curation required to correctly link each component of the metabolic network to external databases.

The ability to correctly match metabolites when comparing reactions is influenced by the different decisions the curators of the pathway databases have taken. For example, in Recon 1 and HumanCyc the protonation state of a metabolite is determined at a pH level of 7.2 and 7.3, respectively. The other three databases always use the neutral form of a metabolite. As illustrated in the C_2_Card centered at the *CTPS* gene (Figure 1), this leads to a reaction mismatch between EHMN and KEGG that have chosen for ammonia (NH_3_) and Recon 1 that has chosen ammonium. The gene and EC number perspectives offer a possible way to uncover such differences. The C_2_Cards application provides an additional means to uncover reactions that are similar, but not an exact match, by allowing the user to specify that one or more mismatches are allowed when querying for a reaction. An example of the results of a query in which one mismatch was allowed is given in Table 2. In this example the reactions only differ in the level of detail with which the metabolite ornithine was described. Note also that the genes and EC number do match, which in this case supports the notion that the two reactions can be considered equivalent. Allowing mismatches also makes it possible to retrieve reactions for which the identity of one or more metabolites could not be established, because of missing identifiers or for which matching on name was hindered by the use of different synonyms.

**Table 2:** **Excerpt of the C**_**2**_**Card centered at the reaction ‘l-arginine + H**_**2**_**O → ornithine + urea’**

**Database**	**Reaction**	**Overlap (%)**	**EC number**	**Gene**	**Pathway**
*H. sapiens* Recon 1	l-arginine[c] + H_2_O[c]	100	3.5.3.1	ARG1	Urea cycle / amino group metabolism
	→				
	**ornithine**[c] + urea[c]				
	l-arginine[m] + H_2_O[m]	100	3.5.3.1	ARG2	Urea cycle / amino group metabolism
	→				
	**ornithine**[m] + urea[m]				
Reactome	l-arginine[c] + H_2_O[c]	66	3.5.3.1	ARG1	Urea Cycle
	→				
	**l-ornithine**[c] + urea[c]				
	l-arginine[m] + H_2_O[m]	66	3.5.3.1	ARG2	Urea Cycle
	→				
	**l-ornithine**[m] + urea[m]				

One metabolite was allowed not to match in this reaction search. The only difference between the reactions is the use of ornithine versus l-ornithine (both in bold). Note that H_2_O is not taken into account for computing the percentage of overlap. ‘[c]’ stands for cytosol and ‘[m]’ for mitochondrion.

### C_2_Cards interfaces

C_2_Cards can be accessed using common JavaScript-enabled browsers on all major platforms including Windows, Linux, and Apple. A C_2_Card centered at a gene or EC number of interest can be retrieved in a single step. For the reaction perspective two routes are offered, either of which requires three steps. A reaction can be found by entering one or more metabolites or by selecting the pathway it is part of in one of the pathway databases. More detail on how to retrieve a C_2_Card is described on the C_2_Cards website (http://www.molgenis.org/c2cards). Once retrieved, a C_2_Card can also be downloaded for off-line use. In addition, for each database the C_2_Cards for all its genes, EC numbers, and reactions, respectively, can be downloaded in tab-delimited format in a single ZIP file.

Next to the web interface, programming interfaces to R, SOAP (Simple Object Access Protocol), and REST (Representational State Transfer) are provided to enable programmatic querying of the collection of C_2_Cards. One possible application would be to perform computational analyses on each of the pathway databases. A typical example is an enrichment test to prioritize pathways most likely to be affected in a given high-throughput experiment. The differences between pathway databases can be quite large both with respect to content and conceptual differences [4]. For example, the number of pathways, in the five selected human pathway databases ranges from 69 in EHMN to 257 in HumanCyc (see Materials and methods). Consequently, it is to be expected that the choice of a particular pathway database affects the outcome of pathway enrichment analyses [18]. It would, therefore, be advisable to apply analyses to multiple pathway databases to verify the robustness of the results. Specifically, to accommodate pathway enrichment analyses, we provide two additional tables, accessible via the programmatic interfaces only. In these tables the metabolites and genes of each pathway database are linked to the corresponding pathways. The results of our reaction comparison could be used to zoom into the outcomes of an enrichment analysis to see if the differences found can perhaps be attributed to the different pathway definitions used by the databases.

Another additional feature offered is the possibility to look up the fate of a metabolite, contained in any of the five databases, by retrieving the list of reactions in which the metabolite of interest participates. Furthermore, databases in which the metabolite is a ‘dead-end’, *i.e.*, it is either only produced or consumed, are explicitly indicated. The list of reactions provided allows the user to find candidate reactions to resolve these dead-ends in the network of a particular database using information from other databases. All reactions in this list are linked to their corresponding C_2_Card.

#### C_2_Cards case studies

For each of the three perspectives we provide a concrete example derived from C_2_Cards^Human^ of consensus and conflicts between the five human pathway databases below. The examples have all been chosen from primary metabolic processes, highlighting that conflicts still occur even in well-studied parts of the metabolic network. Moreover, we focused on examples of differences between databases that are not easily resolved and could point either to conflicting information or to complementary information. The case studies illustrate why manual curation remains crucial to resolve contradicting information and to determine in which cases further biochemical experiments are even required to verify what is correct and what is not.

### Case study I: gene perspective

The C_2_Card focused on the *CTPS* gene (Figure 1) shows that the gene is found in all five databases and is linked to the same EC number by each database. However, to which reaction(s) the databases link the gene differs. EHMN and KEGG both link the gene to two reactions, *i.e.*, a glutamine dependent reaction

$$\begin{array}{l} l-\text{glutamine}+\mathrm{ATP}+\mathrm{UTP}+H_{2}O\to l-\text{glutamate}\\ \;+\mathrm{ADP}+\mathrm{CTP}+\text{orthophosphate} \end{array}$$

and an ammonium dependent reaction

$$\begin{array}{l} \text{ammonium}+\mathrm{ATP}+\mathrm{UTP}\to \mathrm{ADP}+\mathrm{CTP}\\ \;+\text{phosphate}+H^{+}. \end{array}$$

Reactome and HumanCyc only link the gene to the glutamine dependent reaction and Recon 1 only to the ammonium dependent reaction. The C_2_Card focused on the glutamine dependent reaction of Reactome (Figure 1) shows that Recon 1 does contain this reaction, but links it only to the *CTPS2* gene and not to *CTPS*. The same observation can be made when starting from the EC number perspective, as both genes are linked to the same EC number (not shown).

The products of both the *CTPS* and *CTPS2* gene contain a glutamine amidotransferase domain and have high sequence similarity. This and the fact that both gene products have the same EC number suggests that they have similar catalytic activity. For *L. lactis* it is known that both ammonium derived from the hydrolysis of glutamine by the CTP synthase enzymes themselves and ammonium from other external sources of amine donors can be utilized for CTP synthesis [19]. The human counterparts of these enzymes may follow the same reaction mechanism as found for *L. lactis*. This is supported by the fact that under room temperature glutamine is unstable and will dissociate into an ammonium ion and oxo-proline. One could, therefore, hypothesize that *CTPS* and *CTSP2* should be linked to both reactions for *H. sapiens* as well. The glutamine and ammonium dependent activity of *CTPS2* have indeed recently been shown in human embryonic kidney cells [20]. This means that Recon 1 could be improved by linking the glutamine dependent reaction to *CTPS* and the ammonium dependent reaction to *CTPS2*. In Reactome and HumanCyc the ammonium dependent reaction then needs to be added to both genes. In this case study a possible source of confusion for database curators might have been the description given by NC-IUBMB for the EC number (EC:6.3.4.2) assigned to the two gene products. The reaction linked to the EC number is

$$\mathrm{ATP}+\mathrm{UTP}+NH_{3}=\mathrm{ADP}+\text{phosphate}+\mathrm{CTP}$$

and in the comments field it is stated that “Glutamine can replace NH_3_”. This might explain the inconsistencies at the reaction level to some extent.

### Case study II: EC number perspective

**Figure 4:**
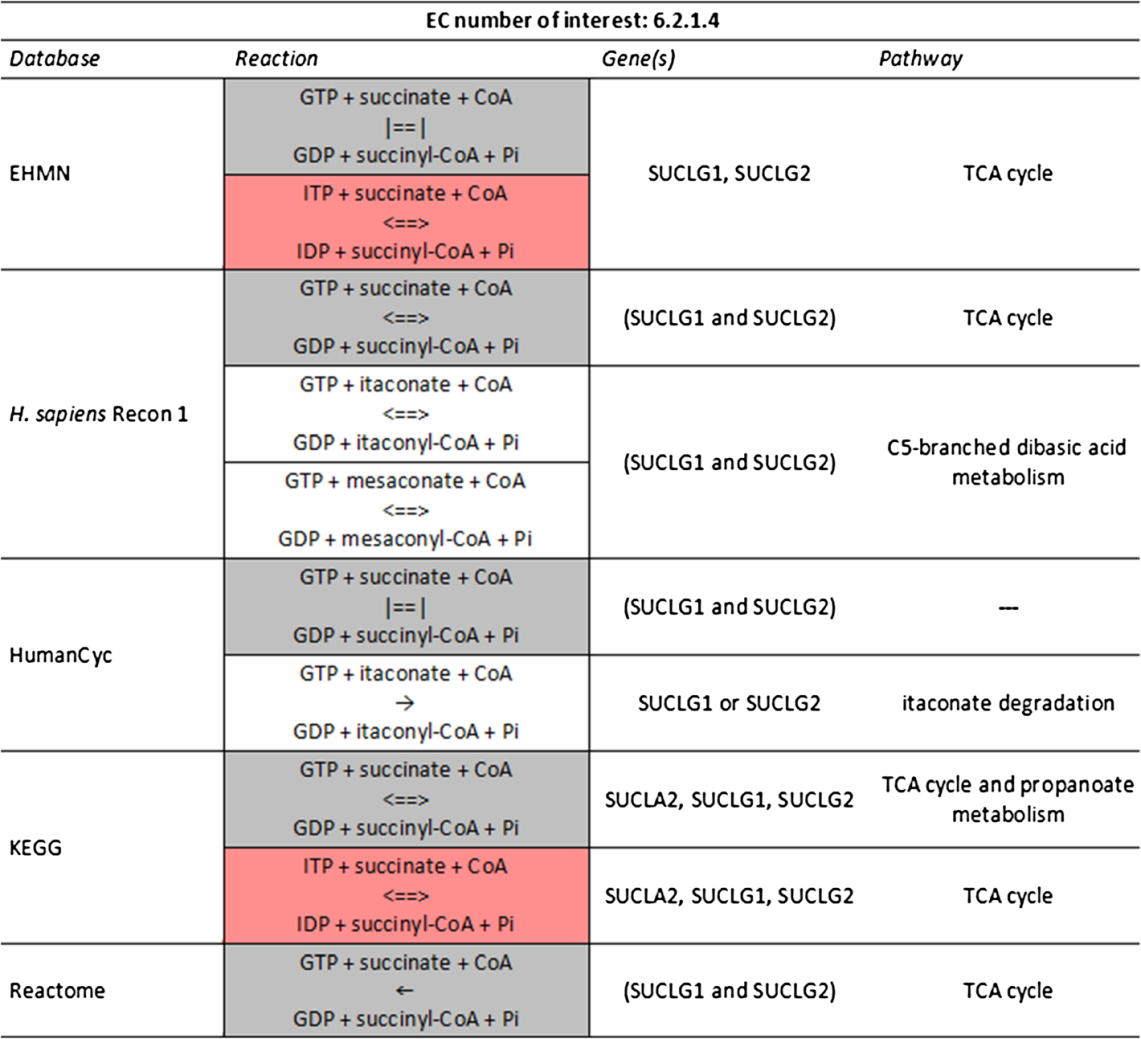
**Excerpt of the C**_**2**_**Card centered at the EC number ‘6.2.1.4’ (succinate-CoA ligase (GDP-forming)).** The reaction in grey is found in all databases, the reaction in red only in EHMN and KEGG. ‘|==|’ indicates no direction provided by the database. Genes are represented by HGNC symbols, retrieved via Entrez Gene IDs. Genes, the products of which form a complex, are placed between parentheses and connected by the Boolean operator ‘and' (see Materials and methods). If gene products are isozymes ‘or’ is used.

The EC number 6.2.1.4 (succinate-CoA ligase (GDP-forming)) is found in all five databases. They all agree on one reaction and two genes linked to it (Figure 4, reaction indicated in grey). The reaction is considered to be part of the tricarboxylic acid (TCA) cycle, a mitochondrial pathway, by all databases except HumanCyc. Both EHMN and KEGG also include a very similar reaction (Figure 4, reaction indicated in red), which only differs with respect to its co-substrates, *i.e.*, IDP/ITP instead of GDP/GTP. Although IDP is a substrate for this enzyme in vitro, it is extremely unlikely to play a role in vivo. The concentrations of IDP and ITP are very low as compared to other nucleotides due to the activity of ITPase. Even if there is a mutation in the ITPA gene, the residual activity of ITPase is still considerable and the IDP/ITP concentrations remain undetectable [21]. Concluding, the reaction with IDP/ITP as co-substrates should not be included in the description of the human metabolic network. Note that also in this case study the description given by NC-IUBMB for this EC number may have been a source of confusion. In the comments field it is stated that ITP can act instead of GTP, which may be true for other organisms, but not for *H. sapiens*.

### Case study III: reaction perspective

All five databases contain the reaction

$$\begin{array}{l} \text{deoxyuridine}+\text{phosphate}<==>\\ \;2-\text{deoxy}-d-\text{ribose}1-\text{phosphate}+\text{uracil} \end{array}$$

and assigned it to similarly named pathways (Table 3). However, there is little agreement on the EC number. Only the one chosen by HumanCyc fits this reaction, however in this database no gene is linked to the reaction. There is no consensus between the databases regarding the genes. For *UPP2* there is clear experimental evidence that its gene product catalyzes the reaction [22]. The activity of the enzymes encoded by *UPP1* and *TYMP* has been evaluated in human liver and placenta [23]. The product of *UPP1* showed some activity towards catalyzing this reaction in placenta. However, no activity was measured in liver, where the enzyme fulfills its main function, the phosphorylation of uridine. The product of *TYMP* mainly functions as a thymidine phosphorylase*.* Activity has been measured for catalyzing the deoxyuridine reaction in liver and to a lesser extent in placenta. For *PNP* there is not enough evidence clearly confirming or refuting that its product can catalyze this specific reaction. Additional experiments are required to determine whether the products of this gene can catalyze this reaction. This also illustrates that even though the majority of the databases links *PNP* to the reaction, this is not necessarily corroborated by conclusive evidence. We can conclude that EHMN, HumanCyc and KEGG should at least link the *UPP2* gene to this reaction. This would resolve the ‘missing gene’ issue in HumanCyc. Furthermore, *TYMP* may need to be added to Recon 1 and HumanCyc. Also *UPP1* might need to be added to Recon 1, HumanCyc and KEGG. Note also that the majority of the databases does not link *UPP2* to this reaction, although clear evidence for its is available.

**Table 3:** **Excerpt of the C**_**2**_**Card centered at the reaction ‘deoxyuridine + phosphate < == > 2-deoxy-d-ribose 1-phosphate + uracil’**

**Database**	**EC number**	**Gene(s)**	**Pathway**
EHMN	2.4.2.1, 2.4.2.4	PNP*, TYMP*, UPP1	Pyrimidine metabolism
*H. sapiens* Recon 1	—	PNP* or UPP2	Nucleotides
HumanCyc	2.4.2.23	—	—
	—	—	salvage pathways of pyrimidine deoxyribonucleotides
KEGG	2.4.2.1	PNP*	Pyrimidine metabolism
	2.4.2.4	TYMP*	
Reactome	2.4.2.3	UPP1 or UPP2	Pyrimidine catabolism and Pyrimidine salvage reactions
	2.4.2.-	TYMP*	

Genes are represented by the HGNC symbol to which their Entrez Gene IDs are linked. The genes on which the majority of the five pathway databases agree, *i.e.*, *PNP* and *TYMP*, are indicated with a ‘*’.

## Discussion

We proposed the concept of Consensus and Conflict Cards to provide concise overviews of the knowledge contained in metabolic pathway databases for an organism of interest. In a single step one can find, for example, a gene of interest and see if the databases agree on the role of its product in the metabolic network. The C_2_Cards will increase the awareness of the differences that exist between the various pathway databases. Other initiatives also provide a web-based interface to browse and search multiple pathway databases [24,25]. However, they are focused on the union of various (pathway) databases instead of explicitly pointing out the differences between pathway databases. Furthermore, they do not provide a clear and compact overview of the content of each of the five selected databases as a C_2_Card does. Also, the C_2_Cards application enables users to find reactions that are similar to the reaction of interest, but that are not exactly the same. The three perspectives offered by the C_2_Cards application provide complementary views on the knowledge contained in the pathway databases. This makes it possible to distinguish differences that reflect a disagreement on the underlying biology (case studies I-III) from differences that may be explained by, for example, different decisions taken on how to represent knowledge (Table 2).

Ultimately, to reconcile differences and to integrate the networks manual curation is required. While a C_2_Card can highlight differences between databases, it cannot distinguish between errors in one (or more) of the databases and cases where databases do not agree due to lack of consensus in the scientific literature. Moreover, for any given organism metabolic pathway databases are still being refined, expanded, and corrected. This makes it challenging to distinguish complementary information from cases in which the database curators purposely excluded, for example, a reaction or gene. Even the parts the pathway databases agree on may need to be reviewed as the databases share information sources and may copy data from each other, thereby possibly propagating incorrect information. Manual curation is also needed to unambiguously assign identifiers to genes and metabolites.

In summary, C_2_Cards offer an elegant solution to bring cases that deserve further inspection to the attention of pathway database curators. The overviews may also point out controversial biological knowledge that should be subject of further research.

## Conclusions

A biologically accurate and complete description of the metabolic network for human and other organisms is of utmost importance to, *e.g.*, increase our knowledge about pathways perturbed by a disease, find new drug targets, and interpret the deluge of high-throughput data. A crucial step towards a more complete description is to combine the knowledge captured by each of the available pathway databases for a specific organism. Much time and effort has already been put into pathway databases and we should profit from this to the fullest extent. However, it requires the commitment and the support of a broad community to construct an initial consensus network and to extend it with new knowledge from domain experts, the scientific literature, and as captured by the various pathway databases. C_2_Cards can contribute to such an endeavor in several ways. As illustrated by the three case studies the C_2_Cards are a perfect starting point for further manual curation of the human metabolic network in future reconstruction jamborees [6]. Our application could be extended in several ways. For example, to support reconstruction efforts, we could indicate whether a reaction is balanced or not, in addition to the already available tool to look up dead-end metabolites. Another possible extension is to further expand the set of five pathway databases currently contained in C_2_Cards^Human^ with additional pathway databases. Importantly, the C_2_Cards application can be set up for other organisms as well (see http://www.molgenis.org/c2cards for a description). Extending each of the three perspectives offered by the C_2_Cards^Human^ to multiple organisms could enable using knowledge about metabolism in model organisms to resolve conflicts between the human pathway databases. Note that this does require the use of an ortholog mapping such as InParanoid [26].

As a guide for integrating pathway databases, we provide overviews of which genes, EC numbers, and reactions can be found in which database. The entries in these overviews are linked to the corresponding C_2_Card. One could start by curating the reactions contained in all or the majority of the databases. In fact, for more than half of the reactions found in all five human metabolic pathway databases, there is no agreement on the EC numbers and genes linked to a reaction [4] and additional curation is needed. C_2_Cards can also be of use if a consensus network for a given organism has already been established. We envision that the C_2_Cards application could serve as a central platform in which the consensus network can be further refined and extended with knowledge available in pathway databases not used for its construction. We are planning to expand C_2_Cards^Human^ with the community-driven consensus human metabolic network Recon 2 [27], which was published while this article was under review. By including Recon 2 as a point of reference, we can compare this state-of-the-art consensus network with other pathway databases. The overview of all reactions in C_2_Cards^Human^, for example, could be a source of candidates for expanding Recon 2. Bringing the differences between the consensus network and other descriptions to the attention of experts would enable further refinement of Recon 2. As a first step towards such a platform, users can already add comments to a C_2_Card, preferably substantiated by references to the literature. They can subscribe to C_2_Cards of their interest and receive an e-mail when new comments are added. Different or even contradictory views possibly held by contributors can be clearly exposed in this forum set-up. Based on these contributions a team of curators could then decide to incorporate the necessary changes in the consensus network, if enough evidence supports this claim. In the future we could extend the forum by allowing people to rank the contributions to bring to the foreground the forum entries deemed most important and thereby aiding the curators. Notably, as illustrated by case study III, it may lead to the conclusion that further biochemical characterization experiments are required. Since pathway databases are continuously being refined and new information is being added, we could also include the possibility to automatically alert the curators by mailing them updated or additional C_2_Cards.

It is important to actively involve domain experts in this continuous curation process, even though they may only indirectly benefit from contributing to such an effort. To make the barrier to contribute as low as possible, the web interface of the C_2_Cards was designed to be easy to use and suitable for users with different backgrounds. The application can be accessed via smartphones and tablets as well, allowing C_2_Cards to be viewed and discussed nearly anywhere. Furthermore, a C_2_Card can be downloaded for off-line use. The curation of a C_2_Card is done at the level of a single reaction or the metabolic functions of a single gene product. This may lower the threshold for experts to contribute as well and also allows (very) detailed knowledge of just a single step in the metabolic network to be added. One way to stimulate expert contributions would be to make the contribution traceable and citable in the form of ‘nanopublications’ [28]. A nanopublication consists of three parts: a *statement*, *e.g.*, protein X (subject) catalyzes (predicate) reaction Y (object), *conditions* under which the statement holds, *e.g.*, a specific compartment, and *provenance* of the statement, *e.g.*, author and literature. Besides that this provides an incentive for experts to share their knowledge, it is also a way to ensure that contributions of curators are substantiated by references to the literature. We also plan to include in C_2_Cards^Human^ the human metabolic pathways of WikiPathways [29], an open platform in which anyone can contribute a pathway. By incorporating the knowledge from this database we indirectly have a second way in which experts can contribute their knowledge. Ultimately, to reconstruct a biochemical network that closely resembles the metabolism of a target organism, extensive literature research and additional biochemical experiments will be needed to resolve all conflicts revealed and to fill in the gaps that remain. The continuous support, time and effort of a large and diverse community are therefore essential. C_2_Cards can contribute to this endeavor by simplifying the identification of consensus and conflicts between pathway databases and lowering the threshold for experts to contribute.

## Materials and methods

### Materials

C_2_Cards^Human^ was built upon the same dataset we used previously [4] for a comparison of five pathway databases, *i.e.*, EHMN, *H. sapiens* Recon 1, HumanCyc, and the human metabolic subsets of KEGG and Reactome (Table 4). For each reaction we retrieved: the EC number(s) and gene(s) linked to it, and the pathway(s) the reaction is part of (Table 5). To compare the reactions, we retrieved for each metabolite, besides its primary name and available synonyms, the chemical formula and the following five types of metabolite identifiers, if available in the specific pathway database: KEGG Compound, KEGG Glycan, PubChem, ChEBI and CAS. There are two types of PubChem IDs, Substance and Compound. Substance IDs are specific for the depositor of the metabolite. Compound IDs unite the different Substance IDs for the same metabolite. We used the CID-SID file (http://ftp://ftp.ncbi.nih.gov/pubchem/Compound/Extras/CID-SID.gz) to convert PubChem Substance IDs to PubChem Compound IDs.

**Table 4:** Overview of metabolic pathway databases used

**Database**	**Export formats used**	**Version**	**Downloaded from**
EHMN	Excel	2	http://www.ehmn.bioinformatics.ed.ac.uk/
*H. sapiens* Recon 1	Flat file, SBML	1	http://bigg.ucsd.edu/
HumanCyc	Flat file	15.0	http://biocyc.org/download.shtml
KEGG	Flat file, KGML	58	http://www.kegg.jp/kegg/download/
Reactome	MySQL database	36	http://reactome.org/download/index.html

All data from the pathway databases was downloaded in the first week of May 2011.

**Table 5:** Pathway database content statistics

**Database**	**Number of**			
	***Genes***	***EC numbers***	***Reactions***	***Pathways***
EHMN	2517	981	3893	69
*H. sapiens* Recon 1	1496	647	2617	96
HumanCyc	3586	1249	1785	257
KEGG	1535	760	1635	84
Reactome	1159	375	1175	171

Genes: counts are based on the internal database identifiers and include genes encoding for a component of a protein complex as separate entities. EC numbers: including incomplete EC numbers. Reactions: if reactions only differ in direction and/or compartments they are counted as one. Pathways: counts for HumanCyc and Reactome are based on the lowest level of their pathway hierarchy.

Although not used for comparing metabolites, we also retrieved the InChI and SMILES of metabolites, when provided by the pathway database, as additional information. For the genes we retrieved the Entrez Gene and Ensembl Gene ID, if available. For display and comparison purposes we mapped the Entrez Gene and Ensembl Gene IDs to their corresponding HGNC symbol as provided by the Entrez Gene and Ensembl database, respectively. Both the Entrez Gene ID and the Ensembl Gene ID were not available for 396 genes in HumanCyc. For 106 of these genes the UniProt ID was used to retrieve the Entrez Gene ID and/or Ensembl Gene ID. All out-of-date identifiers and EC numbers were transferred to the current ID/EC number (Additional file 2). If that was not possible the ID or EC number was flagged as being obsolete. All data is made available under the original license terms of the primary databases.

### Methods

#### Data retrieval and storage

We used dedicated in-house scripts to retrieve the data needed for C_2_Cards^Human^ from the five pathway databases and stored these data in a local MySQL database. The database was designed for easy comparison of the genes, EC numbers, and reactions. A second database, optimized for the queries needed for generating the C_2_Cards^Human^ (Additional file 3), was derived from this database. To avoid heavy computations in the web application the second database contains all pairwise matches on gene and metabolite level and the percentage of overlap between every possible pair of reactions. Note that the C_2_Cards themselves are composed on the fly for a given user query.

#### Matching

In C_2_cards^Human^ genes, EC numbers, metabolites and reactions were matched as follows:

**Genes** Two genes were considered to match if they agreed based on the Entrez Gene ID and/or Ensembl Gene ID. In addition, both types of gene identifiers were mapped to the corresponding HGNC symbols. This provides a basis for matching genes that are not linked to the same genome database, *i.e.*, Entrez Gene or Ensembl, via their HGNC symbol. Moreover, we computed the transitive closure of the gene matches. This means that if for a particular gene there was a match between database A and B, *e.g.*, on Entrez Gene ID, and between database B and C on, *e.g*., Ensembl Gene ID then the gene was considered to match between database A and C as well.

**EC numbers** Matching of EC numbers is straightforward except for 71 incomplete EC numbers the five databases have in total. Up to three numbers of the four that make up a complete EC number may be missing. This is indicated by ‘-’, *e.g.*, EC 1.-.-.-. Incomplete EC numbers have an ambiguous meaning [30]. They may indicate that further specification of the enzyme activity is not possible, but also that a complete EC number for the specific enzyme activity is not yet included by NC-IUBMB. To reduce the number of spurious matches, incomplete EC numbers were matched literally, *i.e.*, the ‘-’ was not treated as a wildcard.

**Metabolites** Metabolites were matched based on the KEGG Compound ID, when available. If the KEGG Compound ID was not provided, the metabolites had to match on any of four other identifiers (KEGG Glycan, ChEBI, PubChem Compound or CAS ID) or on name. In the latter case we also required the chemical formula to match. A difference in the number of H atoms when comparing chemical formulae was ignored. Furthermore, matching on names was case-insensitive and spaces and punctuation were ignored. Also for the metabolite matching we computed the transitive closure (see above).

**Reactions** For reactions we determined the percentage of metabolites they agreed upon, respecting the two sides of a reaction, but ignoring the direction of a reaction. We did not consider e^-^, H^+^, H_2_O in matching reactions as with respect to these particular metabolites reactions are not always balanced. In addition, due to the different pH levels under which the reactions are stated in the databases, the e^-^ and H^+^ metabolites may or may not be included in a reaction. Furthermore, we did not take into account the compartmentalization of reactions. The similarity of two reactions was measured by the percentage of overlap:

$$\frac{\left|\mathrm{matching}\;\mathrm{metabolites}\right|}{\max\left(\left|\mathrm{metabolite}s_{R1}\right|,\left|\mathrm{metabolite}s_{R2}\right|\right)}\times 100\%$$

where *R*1 and *R*2 denote the two reactions being compared. Furthermore, we computed the transitive closure for the reaction matches as well (see above).

It depends on the organism and the specific pathway databases included in the C_2_Cards database which IDs can best be used for comparing genes and metabolites. Only a few changes to the code and the original C_2_Cards database scheme are required to use other IDs for matching. A more detailed description of the changes to make is available on our website (http://www.molgenis.org/c2cards).

#### Construction web application

C_2_Cards^Human^ was built using the Molecular Genetics Information Systems (MOLGENIS) toolkit [31]. This software enables bioinformaticians to model a complete web application having rich data structure and user interfaces using a simple and short XML file. From this model, the toolkit automatically generates software in the Java language that provides a basic web user interface (using Freemarker templates, http://www.freemarker.org), and programming interfaces in Java, R, SOAP and REST to the underlying MySQL database. Building on these generated software we used MOLGENIS ‘plug-in’ framework to program in Java and JavaScript extra features that are specific for C_2_Cards^Human^, such as the various search options. The result is installed on a standard Tomcat web server, but can also run ‘standalone’ using the MOLGENIS embedded web server. A local installation of C_2_Cards^Human^ is also available upon request. All code and the database scheme is open source and can be used as a basis for building a C_2_Cards application for other organisms. A manual on how to do this is available on our website (http://www.molgenis.org/c2cards). The code for the C_2_Cards application is available at http://www.molgenis.org/svn/c2cards/trunk/. A copy of the core MOLGENIS project is also required, which is available at http://www.molgenis.org/svn/molgenis/branches/molgenis_c2cards.

#### Representation

Each row in a C_2_Card contains a reaction, the EC number(s), gene(s), and the pathway linked to the reaction, and the name of the source database. If a reaction was assigned to multiple pathways, a separate row is used for each pathway. The metabolites of a reaction are represented by their primary name as indicated by the pathway database. Although not taken into account when matching reactions, the direction of a reaction and the compartment(s) as indicated by the source database are shown in a C_2_Card. If the direction was not provided this is indicated with ‘|==|’. Multiple EC numbers are connected by a comma. Following the convention used in Recon 1, genes of which the products are isozymes are connected by the Boolean operator ‘or’. If the gene products form a complex ‘and’ is used. EHMN and KEGG, however, do not have a syntactic mechanism for describing isozymes nor complexes. Therefore, if multiple genes were linked to a reaction by EHMN and KEGG, they are connected by a comma. Genes are represented by the HGNC symbol retrieved from Entrez Gene. The Entrez Gene ID was, however, not always available for every gene, and the HGNC symbol could not always be retrieved when the Entrez Gene ID was available. In these cases we used, when available, the Ensembl Gene ID to retrieve the HGNC symbol. For 358 genes the HGNC symbol was not available via either gene identifier type. In this case the gene is represented by its Entrez Gene or Ensembl Gene ID, depending on which of these two was available. For 274 genes in HumanCyc these two gene identifiers were also not available and for these cases the internal gene identifier of HumanCyc is used for representation. If multiple HGNC symbols were linked to a gene they are separated by two underscores. Note also that HumanCyc and Reactome may link multiple Entrez Gene IDs to a single gene, which in most cases will also result in multiple HGNC symbols. Similarly, KEGG and Reactome contain genes linked to multiple Ensembl Gene IDs.

## Abbreviations

BiGG: Biochemically, Genetically, and Genomically structured; C2Cards: Consensus and Conflict Cards; EC: Enzyme Commission; EHMN: Edinburgh Human Metabolic Network; HGNC: HUGO Gene Nomenclature Committee; ID: Identifier; KEGG: Kyoto Encyclopedia of Genes and Genomes; KGML: KEGG Markup Language; MOLGENIS: Molecular Genetics Information Systems; NC-IUBMB: Nomenclature Committee of the International Union of Biochemistry and Molecular Biology; REST: Representational State Transfer; SBML: Systems Biology Markup Language; SOAP: Simple Object Access Protocol; TCA: Tricarboxylic Acid.

## Competing interests

The authors declare that they have no competing interests.

## Authors’ contributions

MDS designed the C_2_Cards and developed the web application, C_2_Cards^Human^. TR and MAS extended the MOLGENIS toolkit with dedicated plug-ins for C_2_Cards^Human^. PDM contributed to the design of the web application. IT contributed to interpretation of the case studies. MDS and PDM wrote the manuscript; IT, MAS, and AHCvK helped draft the manuscript; AHCvK and PDM supervised the project. All authors read and approved the final manuscript.

## Supplementary Material

Additional file 1 **Example of a C**_**2**_**Card.** A C_2_Card centered at an EC number may reveal possible alternative substrates, which is one of the sources of conflict between metabolic pathway databases (Stobbe et al., BMC Syst. Biol., 5:165, 2011). The C_2_Card centered at the EC number 1.1.1.35 (3-hydroxyacyl-CoA dehydrogenase) provides an example of this scenario. The C_2_Card was exported to an Excel file via the web application. This file contains, besides the core table of the C_2_Card, also the overview of the reaction comparison, and information on the metabolites, gene(s), and EC number(s) in the C_2_Card. The number of unique reactions, not taking into account compartmentalization, linked to the EC number 1.1.1.35 varies from 2 in HumanCyc and Recon 1 to 62 in EHMN, as shown in the first worksheet. Click here for file

Additional file 2 **Transferred and obsolete identifiers and EC numbers per database.** Number of transferred and obsolete EC numbers, gene, and metabolite identifiers for each of the five pathway databases. Click here for file

Additional file 3 **Database scheme C**_**2**_**Cards**^**Human**^**.** Overview of the tables in the database of C_2_Cards^Human^. Only the three ‘forum_topic’ tables, the overview tables, and the table with the statistics of the comparison of the five human pathway databases are specific for C_2_Cards^Human^. The SQL script needed to generate the database is available at: http://www.molgenis.org/svn/c2cards/trunk/data/c2cardsdb_empty.sql. Click here for file
