# Supplementary material for: Consensus and conflict cards for metabolic pathway databases
Source: BMC Syst Biol. 2013 Jun 26;7:50. doi: 10.1186/1752-0509-7-50 (PMC3703255; doi:10.1186/1752-0509-7-50)
Supplement: Additional file 2 — Transferred and obsolete identifiers and EC numbers per database. Number of transferred and obsolete EC numbers, gene, and metabolite identifiers for each of the five pathway databases. [file 1752-0509-7-50-S2.pdf]

Additional File 2 – Transferred and obsolete identifiers and EC numbers per database

Genes

	Number of Entrez Gene IDs	
Database	transferred	obsolete
EHMN	4	24
H. sapiens Recon 1	10	5
HumanCyc	38	5
KEGG	1	0
Reactome	10	22

	Number of Ensembl Gene IDs
Database	obsolete
EHMN	37
H. sapiens Recon 1	x
HumanCyc	31
KEGG	12
Reactome	35

EC numbers

	Number of EC numbers		
Database	incomplete	transferred	obsolete
EHMN	41	4	1
H. sapiens Recon 1	2	8	1
HumanCyc	34	2	0
KEGG	34	0	0
Reactome	19	3	0

Metabolites

Number of	KEGG Compound			KEGG Glycan		CAS	ChEBI		PubChem Compound	
Database	incorrectly formatted	transferred	obsolete	incorrectly formatted	obsolete	incorrectly formatted	transferred	obsolete	PubChem Substance IDs that do not map to a PubChem Compound ID	obsolete
EHMN	0	28	4	0	0	0	3	0	35	0[b]
H. sapiens Recon 1	8[a]	14	21	50	2	7	x	x	x	1
HumanCyc	0	9	0	x	x	2[a]	1	39	x	106
KEGG	0	0	0	0	0	0	0	0	259	0[b]
Reactome	0	8	1	x	x	x	0	0	12	0[b]

[a] One could not be corrected and was therefore removed
[b] As the CID-SID.gz file from PubChem was used to convert the PubChem Substance IDs to PubChem Compound IDs these are naturally up-to-date.

An 'x' indicates that the particular identifier is not available for this database.
