# Supplementary material for: Consensus and conflict cards for metabolic pathway databases
Source: BMC Syst Biol. 2013 Jun 26;7:50. doi: 10.1186/1752-0509-7-50 (PMC3703255; doi:10.1186/1752-0509-7-50)
Supplement: Additional file 3 — Database scheme C2CardsHuman. Overview of the tables in the database of C2CardsHuman. Only the three ‘forum_topic’ tables, the overview tables, and the table with the statistics of the comparison of the five human pathway databases are specific for C2CardsHuman. The SQL script needed to generate the database is available at: http://www.molgenis.org/svn/c2cards/trunk/data/c2cardsdb_empty.sql. [file 1752-0509-7-50-S3.pdf]

Additional File 3 – Database scheme C_2Cards^{Human}

Overview of the tables in the database of C_2Cards^{Human}. Only the three 'forum_topic' tables, the overview tables and the table with the statistics of the comparison of the five human pathway databases are specific for C_2Cards^{Human}. The SQL script needed to generate the database is available at:
http://www.molgenis.org/svn/c2cards/trunk/data/c2cardsdb_empty.sql.

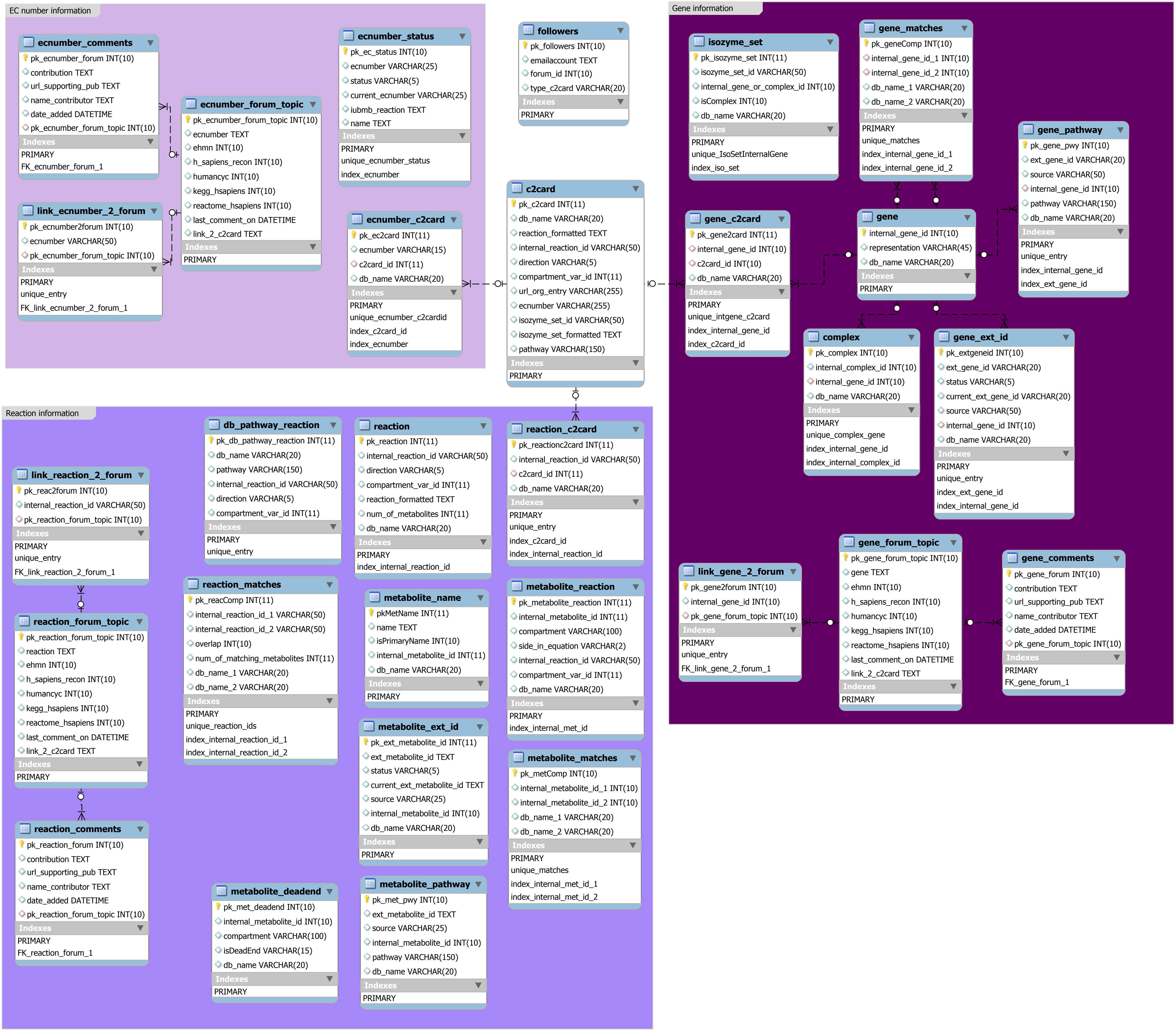
EC number information
ecnumber_comments
pk_ecnumber_forum INT(10)
contribution TEXT
url_supporting_pub TEXT
name_contributor TEXT
date_added DATETIME
pk_ecnumber_forum_topic INT(10)
Indexes
PRIMARY
FK_ecnumber_forum_1
ecnumber_forum_topic
pk_ecnumber_forum_topic INT(10)
ecnumber TEXT
ehmn INT(10)
h_sapiens_recon INT(10)
humancyc INT(10)
kegg_hsapiens INT(10)
reactome_hsapiens INT(10)
last_comment_on DATETIME
link_2_c2card TEXT
Indexes
PRIMARY
ecnumber_status
pk_ec_status INT(10)
ecnumber VARCHAR(25)
status VARCHAR(5)
current_ecnumber VARCHAR(25)
iubmb_reaction TEXT
name TEXT
Indexes
PRIMARY
unique_ecnumber_status
index_ecnumber
link_ecnumber_2_forum
pk_ecnumber2forum INT(10)
ecnumber VARCHAR(50)
pk_ecnumber_forum_topic INT(10)
Indexes
PRIMARY
unique_entry
FK_link_ecnumber_2_forum_1
ecnumber_c2card
pk_ec2card INT(11)
ecnumber VARCHAR(15)
c2card_id INT(11)
db_name VARCHAR(20)
Indexes
PRIMARY
unique_ecnumber_c2cardid
index_c2card_id
index_ecnumber
followers
pk_followers INT(10)
emailaccount TEXT
forum_id INT(10)
type_c2card VARCHAR(20)
Indexes
PRIMARY
c2card
pk_c2card INT(11)
db_name VARCHAR(20)
reaction_formatted TEXT
internal_reaction_id VARCHAR(50)
direction VARCHAR(5)
compartment_var_id INT(11)
url_org_entry VARCHAR(255)
ecnumber VARCHAR(255)
isozyme_set_id VARCHAR(50)
isozyme_set_formatted TEXT
pathway VARCHAR(150)
Indexes
PRIMARY
Gene information
isozyme_set
pk_isozyme_set INT(11)
isozyme_set_id VARCHAR(50)
internal_gene_or_complex_id INT(10)
isComplex INT(10)
db_name VARCHAR(20)
Indexes
PRIMARY
unique_IsoSetInternalGene
index_iso_set
gene_matches
pk_geneComp INT(10)
internal_gene_id_1 INT(10)
internal_gene_id_2 INT(10)
db_name_1 VARCHAR(20)
db_name_2 VARCHAR(20)
Indexes
PRIMARY
unique_matches
index_internal_gene_id_1
index_internal_gene_id_2
gene_pathway
pk_gene_pwy INT(10)
ext_gene_id VARCHAR(20)
source VARCHAR(50)
internal_gene_id INT(10)
pathway VARCHAR(150)
db_name VARCHAR(20)
Indexes
PRIMARY
unique_entry
index_internal_gene_id
index_ext_gene_id
gene_c2card
pk_gene2card INT(11)
internal_gene_id INT(10)
c2card_id INT(10)
db_name VARCHAR(20)
Indexes
PRIMARY
unique_intgene_c2card
index_internal_gene_id
index_c2card_id
gene
internal_gene_id INT(10)
representation VARCHAR(45)
db_name VARCHAR(20)
Indexes
PRIMARY
complex
pk_complex INT(10)
internal_complex_id INT(10)
internal_gene_id INT(10)
db_name VARCHAR(20)
Indexes
PRIMARY
unique_complex_gene
index_internal_gene_id
index_internal_complex_id
gene_ext_id
pk_extgeneid INT(10)
ext_gene_id VARCHAR(20)
status VARCHAR(5)
current_ext_gene_id VARCHAR(20)
source VARCHAR(50)
internal_gene_id INT(10)
db_name VARCHAR(20)
Indexes
PRIMARY
unique_entry
index_ext_gene_id
index_internal_gene_id
link_gene_2_forum
pk_gene2forum INT(10)
internal_gene_id INT(10)
pk_gene_forum_topic INT(10)
Indexes
PRIMARY
unique_entry
FK_link_gene_2_forum_1
gene_forum_topic
pk_gene_forum_topic INT(10)
gene TEXT
ehmn INT(10)
h_sapiens_recon INT(10)
humancyc INT(10)
kegg_hsapiens INT(10)
reactome_hsapiens INT(10)
last_comment_on DATETIME
link_2_c2card TEXT
Indexes
PRIMARY
gene_comments
pk_gene_forum INT(10)
contribution TEXT
url_supporting_pub TEXT
name_contributor TEXT
date_added DATETIME
pk_gene_forum_topic INT(10)
Indexes
PRIMARY
FK_gene_forum_1
Reaction information
link_reaction_2_forum
pk_reac2forum INT(10)
internal_reaction_id VARCHAR(50)
pk_reaction_forum_topic INT(10)
Indexes
PRIMARY
unique_entry
FK_link_reaction_2_forum_1
reaction_forum_topic
pk_reaction_forum_topic INT(10)
reaction TEXT
ehmn INT(10)
h_sapiens_recon INT(10)
humancyc INT(10)
kegg_hsapiens INT(10)
reactome_hsapiens INT(10)
last_comment_on DATETIME
link_2_c2card TEXT
Indexes
PRIMARY
reaction_comments
pk_reaction_forum INT(10)
contribution TEXT
url_supporting_pub TEXT
name_contributor TEXT
date_added DATETIME
pk_reaction_forum_topic INT(10)
Indexes
PRIMARY
FK_reaction_forum_1
db_pathway_reaction
pk_db_pathway_reaction INT(11)
db_name VARCHAR(20)
pathway VARCHAR(150)
internal_reaction_id VARCHAR(50)
direction VARCHAR(5)
compartment_var_id INT(11)
Indexes
PRIMARY
unique_entry
reaction_matches
pk_reacComp INT(11)
internal_reaction_id_1 VARCHAR(50)
internal_reaction_id_2 VARCHAR(50)
overlap INT(10)
num_of_matching_metabolites INT(11)
db_name_1 VARCHAR(20)
db_name_2 VARCHAR(20)
Indexes
PRIMARY
unique_reaction_ids
index_internal_reaction_id_1
index_internal_reaction_id_2
metabolite_deadend
pk_met_deadend INT(10)
internal_metabolite_id INT(10)
compartment VARCHAR(100)
isDeadEnd VARCHAR(15)
db_name VARCHAR(20)
Indexes
PRIMARY
reaction
pk_reaction INT(11)
internal_reaction_id VARCHAR(50)
direction VARCHAR(5)
compartment_var_id INT(11)
reaction_formatted TEXT
num_of_metabolites INT(11)
db_name VARCHAR(20)
Indexes
PRIMARY
index_internal_reaction_id
metabolite_name
pkMetName INT(11)
name TEXT
isPrimaryName INT(10)
internal_metabolite_id INT(11)
db_name VARCHAR(20)
Indexes
PRIMARY
metabolite_ext_id
pk_ext_metabolite_id INT(11)
ext_metabolite_id TEXT
status VARCHAR(5)
current_ext_metabolite_id TEXT
source VARCHAR(25)
internal_metabolite_id INT(10)
db_name VARCHAR(20)
Indexes
PRIMARY
metabolite_pathway
pk_met_pwy INT(10)
ext_metabolite_id TEXT
source VARCHAR(25)
internal_metabolite_id INT(10)
pathway VARCHAR(150)
db_name VARCHAR(20)
Indexes
PRIMARY
reaction_c2card
pk_reactionc2card INT(11)
internal_reaction_id VARCHAR(50)
c2card_id INT(11)
db_name VARCHAR(20)
Indexes
PRIMARY
unique_entry
index_c2card_id
index_internal_reaction_id
metabolite_reaction
pk_metabolite_reaction INT(11)
internal_metabolite_id INT(11)
compartment VARCHAR(100)
side_in_equation VARCHAR(2)
internal_reaction_id VARCHAR(50)
compartment_var_id INT(11)
db_name VARCHAR(20)
Indexes
PRIMARY
index_internal_met_id
metabolite_matches
pk_metComp INT(10)
internal_metabolite_id_1 INT(10)
internal_metabolite_id_2 INT(10)
db_name_1 VARCHAR(20)
db_name_2 VARCHAR(20)
Indexes
PRIMARY
unique_matches
index_internal_met_id_1
index_internal_met_id_2

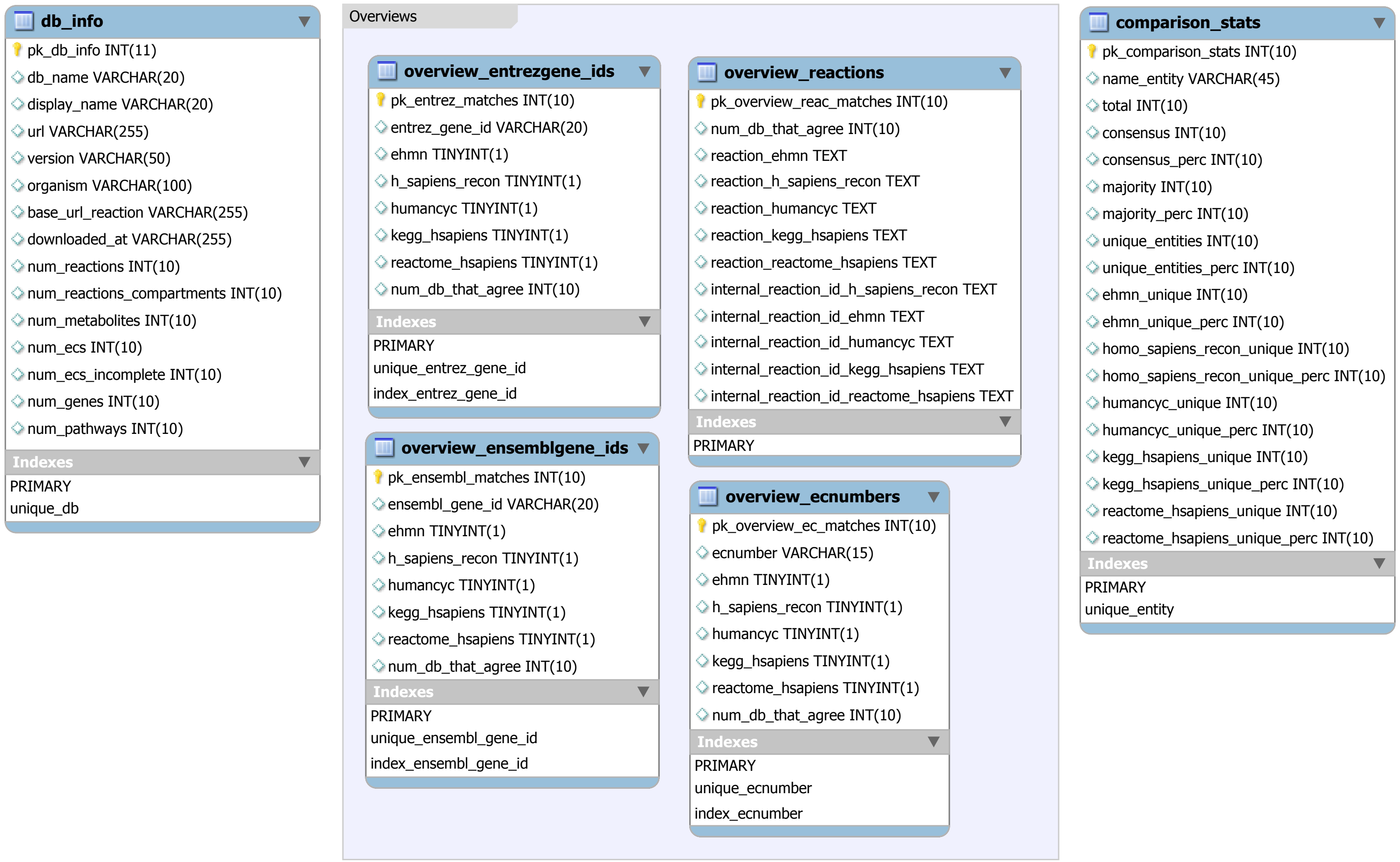

db_info
pk_db_info INT(11)
db_name VARCHAR(20)
display_name VARCHAR(20)
url VARCHAR(255)
version VARCHAR(50)
organism VARCHAR(100)
base_url_reaction VARCHAR(255)
downloaded_at VARCHAR(255)
num_reactions INT(10)
num_reactions_compartments INT(10)
num_metabolites INT(10)
num_ecs INT(10)
num_ecs_incomplete INT(10)
num_genes INT(10)
num_pathways INT(10)
Indexes
PRIMARY
unique_db
Overviews
overview_entrezgene_ids
pk_entrez_matches INT(10)
entrez_gene_id VARCHAR(20)
ehmn TINYINT(1)
h_sapiens_recon TINYINT(1)
humancyc TINYINT(1)
kegg_hsapiens TINYINT(1)
reactome_hsapiens TINYINT(1)
num_db_that_agree INT(10)
Indexes
PRIMARY
unique_entrez_gene_id
index_entrez_gene_id
overview_reactions
pk_overview_reac_matches INT(10)
num_db_that_agree INT(10)
reaction_ehmn TEXT
reaction_h_sapiens_recon TEXT
reaction_humancyc TEXT
reaction_kegg_hsapiens TEXT
reaction_reactome_hsapiens TEXT
internal_reaction_id_h_sapiens_recon TEXT
internal_reaction_id_ehmn TEXT
internal_reaction_id_humancyc TEXT
internal_reaction_id_kegg_hsapiens TEXT
internal_reaction_id_reactome_hsapiens TEXT
Indexes
PRIMARY
overview_ensemblgene_ids
pk_ensembl_matches INT(10)
ensembl_gene_id VARCHAR(20)
ehmn TINYINT(1)
h_sapiens_recon TINYINT(1)
humancyc TINYINT(1)
kegg_hsapiens TINYINT(1)
reactome_hsapiens TINYINT(1)
num_db_that_agree INT(10)
Indexes
PRIMARY
unique_ensembl_gene_id
index_ensembl_gene_id
overview_ecnumbers
pk_overview_ec_matches INT(10)
ecnumber VARCHAR(15)
ehmn TINYINT(1)
h_sapiens_recon TINYINT(1)
humancyc TINYINT(1)
kegg_hsapiens TINYINT(1)
reactome_hsapiens TINYINT(1)
num_db_that_agree INT(10)
Indexes
PRIMARY
unique_ecnumber
index_ecnumber
comparison_stats
pk_comparison_stats INT(10)
name_entity VARCHAR(45)
total INT(10)
consensus INT(10)
consensus_perc INT(10)
majority INT(10)
majority_perc INT(10)
unique_entities INT(10)
unique_entities_perc INT(10)
ehmn_unique INT(10)
ehmn_unique_perc INT(10)
homo_sapiens_recon_unique INT(10)
homo_sapiens_recon_unique_perc INT(10)
humancyc_unique INT(10)
humancyc_unique_perc INT(10)
kegg_hsapiens_unique INT(10)
kegg_hsapiens_unique_perc INT(10)
reactome_hsapiens_unique INT(10)
reactome_hsapiens_unique_perc INT(10)
Indexes
PRIMARY
unique_entity
